# Non-Invasive Cytology Brush PCR Diagnostic Testing in Mucosal Leishmaniasis: Superior Performance to Conventional Biopsy with Histopathology

**DOI:** 10.1371/journal.pone.0026395

**Published:** 2011-10-27

**Authors:** Andrea K. Boggild, Braulio Mark Valencia, Nicolas Veland, Ana Pilar Ramos, Flor Calderon, Jorge Arevalo, Donald E. Low, Alejandro Llanos-Cuentas

**Affiliations:** 1 Tropical Disease Unit, Division of Infectious Diseases, Toronto General Hospital, University of Toronto, Toronto, Canada; 2 Instituto de Medicina Tropical “Alexander von Humboldt”, Universidad Peruana Cayetano Heredia (UPCH), Lima, Peru; 3 Departamento de Bioquimica, Biologia Molecular y Farmacologia, Facultad de Ciencias, Universidad Peruana Cayetano Heredia (UPCH), Lima, Peru; 4 Laboratories Branch, Ontario Agency for Health Protection and Promotion, Etobicoke, Canada; 5 Department of Laboratory Medicine and Pathobiology, University of Toronto, Toronto, Canada; 6 Hospital Nacional Cayetano Heredia, Lima, Peru; The University of Maryland, United States of America

## Abstract

**Background:**

Traditional methods of diagnosing mucosal leishmaniasis (ML), such as biopsy with histopathology, are insensitive and require collection of an invasive diagnostic specimen.

**Methods:**

We compared standard invasive procedures including biopsy histopathology, biopsy PCR, and leishmanin skin test (LST) to a novel, non-invasive, cytology-brush based PCR for the diagnosis of ML in Lima, Peru. Consensus reference standard was 2/4 tests positive, and outcome measures were sensitivity and specificity. *Leishmania* species identification was performed by PCR-based assays of positive specimens.

**Results:**

Twenty-eight patients were enrolled, 23 of whom fulfilled criteria for a diagnosis of ML. Sensitivity and specificity of biopsy with histopathology were 21.7% [95% CI 4.9–38.5%] and 100%; 69.6% [95% CI 50.8–88.4%] and 100% for LST; 95.7% [95% CI 87.4–100%] and 100% for biopsy PCR; and 95.7% [95% CI 87.4–100%] and 90% [95% CI 71.4–100%] for cytology brush PCR using both Cervisoft® and Histobrush® cervical cytology brushes. Represented species identified by PCR-RFLP included: *L. (V). braziliensis* (n = 4), and *L. (V). peruviana* (n = 3).

**Conclusions:**

Use of commercial grade cytology brush PCR for diagnosis of ML is sensitive, rapid, well tolerated, and carries none of the risks of invasive diagnostic procedures such as biopsy. Further optimization is required for adequate species identification. Further evaluation of this method in field and other settings is warranted.

## Introduction

Mucosal leishmaniasis (ML) is a severe and stigmatizing chronic sequela of infection with predominantly New World species of *Leishmania* including *L. (Viannia) braziliensis* [1]–[5]. Along with Brazil and Bolivia, Peru contributes more than 90% of ML cases worldwide [1]. Differentiating ML from other endemic etiologies such as tuberculosis, non-tuberculous mycobacterial infections, rhinoscleroma, paracoccidioidomycosis, and malignancy is difficult on clinical grounds as manifestations such as nasal injection, pruritus, and infiltration, epistaxis, dysphonia, and palatal infiltration may be common to all. Coupled with the highly toxic nature of standard antimonial therapy, the broad differential diagnosis of mucosal lesions in Peruvian patients necessitates the use of accurate diagnostic modalities.

Traditional methods of diagnosing ML, such as biopsy with histopathology, are insensitive and require collection of an invasive diagnostic specimen [2], [6]. Invasive specimen collection is difficult to perform in remote under-resourced settings, and without technical expertise [7]–[9]. While PCR is a highly sensitive technique and is quickly becoming a favored ‘gold standard’ for the diagnosis of leishmaniasis [6], [10]–[14], this platform has mostly been used on invasive diagnostic specimens in ML such as biopsies [2], [9], [14]. Accurate diagnosis in the absence of a well-performing gold standard is an ongoing challenge in leishmaniasis [15]. There is therefore a need for sensitive, accurate non-invasive diagnostic testing in ML.

We herein compared several ‘traditional’ methods for diagnosing ML including biopsy with histopathology, biopsy PCR, and leishmanin skin test (LST) to the novel, non-invasive method of cytology brush PCR using 2 different commercial grade cervical cytology brushes. In addition, we performed species identification using PCR-based assays of clinical specimens, which is important in countries like Peru where several members of the *Leishmania (Viannia)* subgenus can cause mucosal disease.

## Methods

### Ethics Statement

This study was approved by the Institutional Review Boards of Hospital Nacional Cayetano Heredia (HNCH) and the University of Toronto. All patients provided written informed consent for the study procedures prior to enrolment.

### Study Site

The study was conducted at the *Leishmania* Clinic of the Instituto de Medicina Tropical “Alexander Von Humboldt” and HNCH, in Lima, Peru, between January and December 2010. The Institute houses a large outpatient clinic for the diagnosis and management of American tegumentary leishmaniasis, with an average of 30–40 new cases diagnosed per month [7], [16].

### Study Population

Consecutive patients presenting to the *Leishmania* Clinic for the evaluation of mucosal (nasal, buccal, oral, pharyngeal) and/or skin lesions were approached to participate in this study, and screened for eligibility criteria. All patients were interviewed and examined by a clinic physician. Direct anterior rhinoscopy and oropharyngoscopy were performed on all patients (including those referred for cutaneous ulcers), to determine if mucosal abnormalities such as erythema, infiltration, or ulceration were present. We included patients who were referred to the *Leishmania* Clinic for suspected ML or CL; had one or more mucosal lesions with a clinical indication for mucosal biopsy; and were able to give informed consent for the diagnostic procedures. We excluded patients undergoing active treatment for ML or CL, and those with any contraindication to mucosal biopsy.

### Sampling

#### Cytology brushes

**Figure 1:**
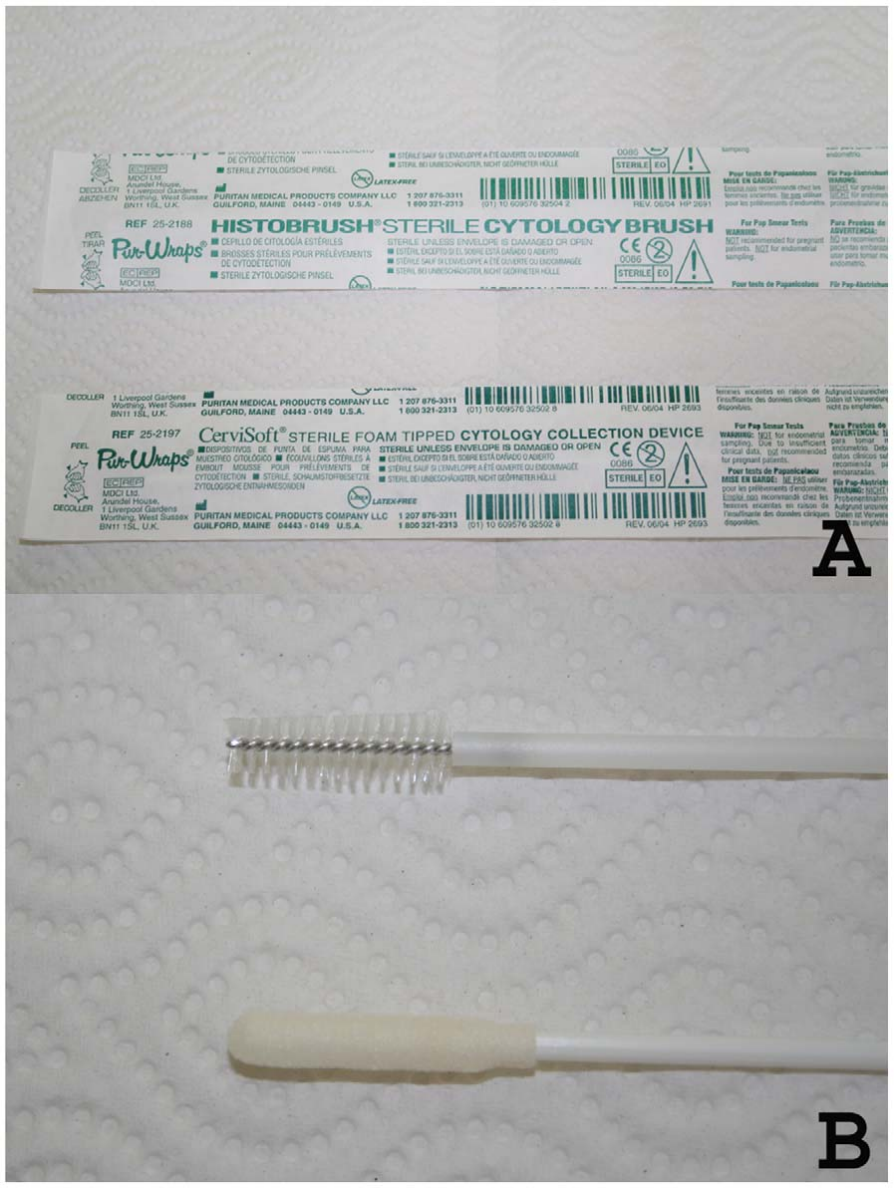
Cervical cytology brushes. A, sterile wrapped CerviSoft® and Histobrush® cervical cytology brushes; B, CerviSoft® and Histobrush® cytology brush tips.

After removing any overlying scab or crust with moistened gauze, and cleansing the mucosal lesion with isopropyl alcohol, sterile and duplicate CerviSoft® (Puritan Medical Products, Maine) and Histobrush® (Puritan Medical Products, Maine) cervical cytology brushes (Figure 1) were rolled clockwise on the lesion 5 times each in sequence. Each cytology brush has a cylindrical handle of several inches in length, and a foam or bristled tip of 1-inch in length for collection of clinical specimens (Figure 1). Cytology brush tips were then cut off with sterile scissors directly into 1.5-mL Eppendorf tubes containing 700 µL 100% ethanol and stored at −20 C for qualitative PCR testing. Control nasal septum and buccal cytology brush specimens were collected and processed as above from 5 healthy volunteers with normal mucosa living in a non-endemic area.

#### Mucosal biopsy

After collection of the cytology brush specimens and cleansing the lesion again with isopropyl alcohol, mucosal lesions were anesthetized with 20 mg/mL lidocaine spray. Two small biopsy specimens were then obtained from lesions using sterile nasal or ethmoid biopsy forceps. The tissue was then stored in 1.5-mL Eppendorf tubes containing 700 µL 100% ethanol at −20°C for qualitative PCR testing, or placed in 10% formalin for histopathology with hematoxylin and eosin, Ziehl-Neelsen, and Giemsa staining. Sterile gauze was applied with pressure to the mucosal lesion until hemostasis was achieved.

### Leishmanin Skin Test

Leishmanin skin tests were applied and read as described [7], [16]. A positive result was indicated by ≥5 mm of erythema and induration as previously described [7], [16], [17].

### Isolation of DNA from Cytology Brushes and Biopsy Specimens

Prior to DNA extraction, samples were centrifuged at 3000 g for 5 min and ethanol was discarded. Biopsied tissues were disaggregated with a sterile scalpel. Disaggregated tissue and cytology brushes were processed for DNA isolation using the High Pure PCR Template Preparation Kit® (Roche, Mannheim, Germany) according to manufacturer's instructions.

### Kinetoplastid DNA (kDNA) Polymerase Chain Reaction

*Leishmania* kDNA PCR was performed using the HotStar Taq Plus DNA Polymerase kit (QIAGEN, Hilden, Germany) and conditions were as described [7]. Two pairs of primers were used as previously described [7], [18]. Amplicons were visualized on 3% agarose gels (Promega, Madrid, Spain) and stained with ethidium bromide.

### Species Identification by PCR and Restriction Fragment Length Polymorphism (PCR-RFLP) of Genomic Targets

Four PCR assays targeting different sequences specific to *Leishmania* sub-genus *Viannia* species including *L. (V.) braziliensis*, *L. (V.) peruviana*, and *L. (V.) guyanensis*, the principal causative species in Peru, were used for the species identification following initial kDNA PCR. PCR assays were performed using the HotStar Taq Plus DNA Polymerase kit (QIAGEN, Hilden, Germany) as previously described [7].

The first assay, targeting the mannose phosphate isomerase gene (MPI), consisted of two separate reactions employing allele-specific reverse primers, which distinguish *L. (V.) peruviana* from *L. (V.) braziliensis* and *L. (V.) guyanensis*, as previously described [7], [19]. MPI PCR conditions were as described [7], [19]. The second assay, targeting the cysteine proteinase B (*Cpb*) gene, employed primers which distinguish between *L. (V.) braziliensis* and non-*L. (V.) braziliensis* species as previously described [7], [20], [21]. *Cpb* PCR conditions were as described [7], [20], [21]. The third assay, targeting heat shock protein 70 (*hsp70*), employed primers which distinguish between *L. (V.) guyanensis* and non-*L. (V.) guyanensis* species as previously described [7], [21], [22]. *Hsp70* PCR conditions were as described [21], [22].

A fourth and final PCR assay was used to confirm species on those samples that yielded weak bands or lack of amplification products in previous assays. The PCR target was a 870 bp fragment of *Leishmania* glycoprotein of 63 kDa (*gp63*), with the following primer sequences: MUS (fwd) 5′- GTGGGTGTCATCAACATCCC – 3′ and MUSA3 (rev) 5′- CTGCTGCCGTACACCTGGAC – 3′ [23]. *Gp63* PCR conditions were as follows: 95°C for 5 min, followed by 45 cycles of denaturation at 94°C for 30 s; primer annealing at 63°C for 60 s; extension at 72°C for 60 s, and a final extension step at 72°C for 6 min (iCycler iQ, Bio-Rad). All PCR products were visualized on 1.5% agarose gels (Promega, Madrid, Spain) and stained with ethidium bromide.

### Restriction fragment length polymorphism analysis of *Cpb*, *Hsp70, and gp63* PCR products (PCR-RFLP)

Following *cpb*, *hsp70* and *gp63* PCR amplification as above, products were separately digested overnight at 65°C for the *cpb* assay, or 37°C for the *hsp70* and *gp63* assay, in a total volume of 20 µL, with 5 U of each restriction enzyme. The following enzymes were used in each reaction: *Taq*I (*cpb*) and *Hae*III (*hsp*70) (Fermentas, Burlington, Canada). For *gp63*, products were digested in duplicate. One reaction was digested with *Hinc*II and the second with *Sal*I restriction enzyme (Fermentas, Burlington, Canada). Restriction fragments were then analyzed separately using 2.5% agarose gels for *cpb* and *gp63* or 4% agarose gels for *hsp70* (Promega, Madrid, Spain), and stained with ethidium bromide. When weak amplification product was observed after PCR, restriction fragments were separated using 12% polyacrylamide gel electrophoresis using the MiniProtean III system (Bio-Rad, Hercules, CA, USA), and stained with silver stain (Promega, Madrid, Spain). *Mpi* PCR distinguishes *L. (V.) peruviana*, while *Cpb* PCR-RFLP distinguishes *L. (V.) braziliensis*, and *hsp70* PCR-RFLP differentiates *L. (V.) guyanensis* from *L. (V.) lainsoni*.

### Composite Reference Standard

We defined a lesion as ML when any 2 of 4 tests were positive, where tests refer to biopsy with histopathology; biopsy PCR; LST; or cytology brush PCR. These 4 tests served as the composite reference standard against which each individual diagnostic test was compared. Assessors of LST, histopathology, and PCR were blind to the results of the other assays.

### Sample Size Calculation

Based on existing literature [2], [14], [24]–[26], we estimated the overall sensitivity of gold standard biopsy with histopathology to be 40%, and the sensitivity of biopsy PCR to be 90–95%. In order to achieve a sensitivity of cytology brush PCR better than the gold standard histopathology and comparable to biopsy PCR, assuming an α = 0.05 and a power of 80%, 28 patients were required per group. For sensitivity analysis, the aforementioned composite reference standard was applied, and the unit of analysis was the patient.

### Statistical Analysis

Descriptive statistics (mean, SD, median, range) were calculated for continuous variables, and differences were compared using 2-tailed t-testing. Categorical variables were quantitated by proportions, and differences between the groups were compared using Yate's corrected Chi-square analysis. Differences in sensitivities and specificities were compared using the z-test. Statistical analyses were performed using SigmaStat 2.03 software (SPSS Inc., Chicago, IL). Level of significance was set at p<0.05.

## Results

Twenty-eight patients were enrolled in the study: 23 males and 5 females. Of 28 patients enrolled, 23 were referred for suspicious mucosal lesions only, and 5 were referred for evaluation of cutaneous lesions who were then noted to have mucosal involvement on examination. Clinical and demographic characteristics of the cohort are summarized in table 1. Median age was 48 years (range 16–87 years), and median duration of exposure in the risk area was 7.5 years (range 2 days–75 years). Median duration of illness was 25.5 months (range 1 month–20 years). Of 28 patients enrolled, 15 (54%) had a past history of CL, and 4 (14%) had old scars suspicious for past CL, but no previous definitive diagnosis (Table 1). Of 23 patients diagnosed with ML, 4 had intercurrent cutaneous leishmaniasis.

**Table 1:** Clinical and Demographic Characteristics of 28 Patients with Suspected Mucosal Leishmaniasis.

Age, Sex	Lesion Location	Intercurrent CL*	LST	Histopathology	PCR Biopsy	PCR Brush**
60, M§	NS	No	Pos	Lymphoeosinophilic infiltrate	Pos	Pos
46, M¶	NS	No	Neg	Granulomatous inflammation	Pos	Pos
62, F	NS	No	Neg	Lymphoplasmo-histiocytosis	Pos	Pos
68, M	NS	Yes	Neg	Lymphoeosinophilic infiltrate	Pos	Pos†
66, M§	NS	No	Pos	Parasitic granuloma with amastigotes	Pos	Pos
57, M	Canthus	Yes	Pos	Granulomatous inflammation with amastigotes present	Pos	Pos
36, M	NS	Yes	Pos	Normal cartilage	Pos	Pos
49, F¶	NS	No	Neg	Polymorphonuclear lymphocytic infiltrate	Pos	Pos
75, F	NS	Yes	Pos	Lymphocytic infiltrate	Pos	Pos
16, M	NS	No	Pos	Parasitic granuloma with amastigotes	Pos	Pos
38, M§	Buccal	No	Neg	PAS-positive bodies suspicious for *Paracoccidioides braziliensis* infection	Pos	Pos
31, M§	Hard Palate	No	Neg	Granulomatous inflammation	Pos	Pos
87, M§	Buccal	No	Neg	Epidermoid carcinoma	Neg	Neg
52, M§	NS	No (stasis ulcers)	Pos	Granulomatous inflammation	Pos	Pos
63, M¶	NS	No	Pos	Granulomatous inflammation	Pos	Pos
32, M§	NS	No	Pos	Granulomatous inflammation	Pos	Neg
19, M	NS	No	Neg	Perivascular eosinophilic infiltrate	Neg	Neg
41, M	Hard Palate	No	Pos	Granulomatous inflammation	Pos	Pos
40, M§	NS	No	Neg	Granulomatous inflammation	Neg	Pos
20, F§	NS	No	Pos	Granulomatous inflammation	Pos	Pos
50, M§	NS	No	Pos	Granulomatous inflammation	Neg	Pos
47, M§	NS	No	Pos	Parasitic granuloma with amastigotes	Pos	Pos
62, F¶	NS	No	Neg	Acute and chronic inflammatory infiltrate	Neg	Neg
43, M§	NS	No	Pos	Parasitic granuloma with amastigotes	Pos	Pos
62, M	NS	No	Neg	Granulomatous inflammation	Neg	Neg
35, M§	NS	No	Neg	Granulomatous inflammation	Pos	Pos
55, M§	NS	No	Pos	Granulomatous inflammation	Pos	Pos
23, M§	NS	No	Pos	Epithelial hyperplasia and necrosis	Pos	Pos

*Intercurrent CL confirmed by smear or culture of cutaneous lesion specimens.

**All 5 control volunteers had negative cytology brush specimens.

† CerviSoft® and Histobrush® PCR demonstrated 97% concordance; in 1 patient, CerviSoft® PCR was positive and Histobrush® PCR was negative.

§ Patient with known previous CL on history.

¶ Patient with scars suspicious to be healed CL but no definitive history of diagnosis.

Abbreviations: CL, cutaneous leishmaniasis; F, female; LST, leishmanin skin test; M, male; Neg, negative; NS, nasal septum; Pos, positive.

Performance characteristics of each tested assay are summarized in table 2. Biopsy PCR was 95.7% sensitive [95% CI 87.4–100%], and 100% specific, with pooled cytology brush PCR demonstrating 95.7% [95% CI 87.4–100%] sensitivity and 90% [95% CI 71.4–100%] specificity. Sensitivity and specificity of Cervisoft® brush PCR were 95.7% and 90%, while Histobrush® PCR demonstrated sensitivity of 91.3% [95% CI 79.8–100%], and specificity of 90% [95% CI 71.4–100%]. Compared to cytology brush PCR, traditional biopsy with histopathology and LST had poorer performance characteristics with sensitivities of 21.7% [95% CI 4.9–38.5%] (p<0.001) and 69.6% [95% CI 50.8–88.4%] (p = 0.016), respectively [Table 2]. Expanding the definition of biopsy with histopathological diagnosis to include granulomatous inflammation, rather than strictly to the presence of visible amastigotes, increased the sensitivity of biopsy with histopathology to 71.4% [95% CI 52.9–89.9%], which was still inferior to cytology brush PCR or biopsy PCR (p = 0.024).

**Table 2:** Analysis of 5 Diagnostic Tests used in the Evaluation of 28 Patients with Suspected Mucosal Leishmaniasis.

Assay	Number Positive	Number Negative	Sensitivity (%)	Specificity (%)	PPV (%)	NPV (%)
kDNA PCR of Biopsy Specimen	22	6	95.7	100.0	100.0	83.3
kDNA PCR of CerviSoft® brushes	23	10*	95.7	90.0	95.7	90.0
kDNA PCR of Histobrush® brushes	22	11*	91.3	90.0	95.5	81.8
LST	16	12	69.6	100.0	100.0	41.7
Biopsy with Histopathology	5	23	21.7	100.0	100.0	21.7

*includes nasal and buccal specimens from 5 healthy control volunteers.

Abbreviations: LST, leishmanin skin test; NPV, negative predictive value; PPV, positive predictive value.

Subjective tolerability of the CerviSoft® brush was superior to that of the Histobrush®, as it was reported to cause less discomfort and was softer on the mucosa, particularly in the nose. All healthy control volunteers had negative CerviSoft® and Histobrush® cytology brush PCR.

Of 23 patients with ML, PCR-based assays led to species identification in 7. Represented causative species included: *L. (V.) braziliensis*, 4 patients; and *L. (V.) peruviana*, 3 patients. There were no cases of *L. (V.) guyanensis* or *L. (V.) lainsoni* identified (Table 3).

**Table 3:** Species identification from 2 sets of specimens out of 16 PCR-positive lesions subsequently tested with PCR-based assays targeting the mannose phosphate isomerase, cysteine proteinase B, heat shock protein 70, and gp63 genes.

*Leishmania* Species	Number (% of those tested)
*L. (V.) braziliensis*	4 (25%)
*L. (V.) guyanensis*	0
*L. (V.) peruviana*	3 (19%)
Not identifiable	9 (56%)
Not tested*	12

*Only specimens with sufficient amplifiable DNA from the kDNA PCR of cytology brushes were selected for direct clinical specimen PCR.

## Discussion

We have demonstrated that *Leishmania (Viannia)* kDNA can be detected in non-invasive cytology brush specimens for the diagnosis of ML. PCR of non-invasive cytology brush specimens had a comparable performance to PCR of biopsy specimens, and was superior to either conventional biopsy with histopathology or LST. Compared to biopsy, non-invasive cytology brushes are easier to obtain, require no technical expertise or anesthesia, cause no discomfort to the patient, do not carry risks of bleeding or infection, and obviate the need for sharps, sharps biosafety disposal, or concerns regarding needle stick injuries in the health care worker. Unlike biopsies, cytology brush specimens can be easily collected in the field and transported back to a reference center for testing. Thus, our data represent an advance in the approach to diagnostic testing in ML that will benefit the patient and health care worker alike.

Although PCR of biopsy and brush specimens were highly sensitive and specific, there may have been 1 patient with known previously treated CL (1990) and ML (1992), and new isolated involvement of the buccal mucosa with no other focus of infection, who was a biological false positive. This patient had PAS-positive bodies on histopathology suspicious for *Paracoccidioides braziliensis*, but also had a buccal mucosa biopsy and cytology brushes that were positive for *L. (Viannia)* kDNA by PCR. It is therefore possible that this patient had detectable amounts of persistent parasite DNA in the mucosa and new infection with *Paracoccidioides braziliensis*. Alternatively, the patient may have had active infection with both paracoccidioidomycosis and leishmaniasis. In any case, this patient highlights the need for non-invasive multiplex assays that can differentiate between common causes of mucosal lesions in the tropics. Although the patient fulfilled reference criteria for a diagnosis of ML, the uncertainty surrounding the diagnosis given the histopathology also suggests that further refinements to the cytology brush method, possibly including a quantitative component, are warranted.

*Leishmania* kDNA has been detected in the normal mucosa of Latin American patients with CL [9], which raises the possibility that detectable kDNA in the mucosa does not necessarily reflect ML. While it is possible that patients in our series merely had detectable kDNA in the mucosa, and not necessarily true ML, that they all had clinical evidence of ML (ie, mucosal lesions) and fulfilled a consensus reference standard for diagnosis argues against this possibility. However, in one patient with confirmed CL (by smear and culture of skin lesion aspirate) and erythematous nasal mucosa, a diagnosis of ML was made based on positive nasal biopsy PCR, cytology brush PCR, and LST, despite normal nasal septal histopathology. It is possible that this patient simply had detectable kDNA in the mucosa by multiple means, thus fulfilling reference standard criteria for diagnosis, but no true ML. Given the paucity of data surrounding treatment outcomes on patients like this who have CL and detectable kDNA in the mucosa, we erred on the side of caution and implemented a 28-day ML treatment regimen rather than a 21-day CL regimen. Prospective testing of CL patients with normal mucosa by nasal and buccal cytology brush PCR in our center may be indicated to better inform management of this unique situation. Regardless of how the mucosa is labeled histopathologically, treatment of detectable kDNA in the mucosa of CL patients with a ML regimen is likely warranted until further data, which inform our understanding of mucosal dissemination, are accrued.

Species identification is important in countries like Peru where several members of the *L. Viannia* subgenus are co-endemic and portend different prognoses and response to therapy [4]. *L. (V). braziliensis* is historically the most well represented causative species in ML [2], [4], [5]. We have demonstrated that related species including *L. (V). peruviana* are implicated in mucosal disease as well. Case-report level data implicating non-braziliensis sub-species in ML is also mounting [2], [5], [27], [28]. Further optimization of direct-cytology brush species identification is required. As genomic targets were used for species identification PCR and RFLP assays (rather than the kinetoplast target used for the diagnostic PCR), enhanced collection of higher concentrations of amastigotes or parasite DNA from the lesion by additional revolutions of the brush may improve the yield.

In summary, we have demonstrated that cytology brush PCR using CerviSoft® and Histobrush® cervical cytology brushes is adequate for the diagnosis of ML though identification of causative species requires further optimization. We have further demonstrated its superior performance to the gold standard biopsy with histopathology. Cytology brush PCR offers numerous practical advantages over biopsy PCR including simplicity, tolerability, and cost efficiency due to the lack of need for highly trained personnel to collect the specimen, anesthesia, sterile biopsy instruments, and sharps biohazard disposal and precautions. At just 30–50 cents (US) per cytology brush, this novel diagnostic specimen is practical and comparatively affordable. It can be transported easily to a reference center for diagnostic testing and is likely appropriate for field situations. Future field studies are indicated, as are studies that aim to differentiate between common causes of mucosal lesions in the tropics using this simple non-invasive specimen.
